# Elevation of the Epiglottis

**Published:** 1881-11

**Authors:** 


### ELEVATION OF THE EPIGLOTTIS.

Dr. Howard (New York) read a paper on the Elevation of the Insensitive Epiglottis by Position. He showed that the insensitive epiglottis is elevated instantly, and to the fullest extent, by forcibly extending the head and neck. In doing this, the hyoid bone is carried forwards and upwards, by means of the muscles passing between that bone and the inferior maxilla; and the epiglottis is drawn after the hyoid bone, in consequence of its attachment thereto by the hyo-epiglottic ligament. This is the only way in which the complete elevation of the epiglottis of a patient, in the supine position, and unconscious from anæsthetics or otherwise, can be accomplished. Mere drawing forward of the tongue, and elevation of the lower jaw, has little or no effect upon the epiglottis; but, by throwing back the head as far as possible, and at the same time keeping the shoulders and lower part of the neck elevated, the epiglottis is raised upwards and forwards, so that air freely enters the trachea.—*Proceedings International Congress.*

